# Isolation of F-specific *Inovirus* filamentous phages from environmental sewage samples

**DOI:** 10.1016/j.mex.2025.103701

**Published:** 2025-10-30

**Authors:** Natasha Theriault, Bradley W.M. Cook, Steven S. Theriault, Deborah A. Court

**Affiliations:** a University of Manitoba, Department of Microbiology, Winnipeg, Manitoba, Canada; b Cytophage Technologies Inc., Oak Bluff, Manitoba, Canada

**Keywords:** Filamentous phages, Environmental phage isolation, Turbid plaques, Hazy plaques, Agar overlay, Plaque PCR screening

## Abstract

Bacteriophage (phage) isolation from various environmental sources is an important step in identifying phages suitable for therapeutic and/or research purposes. Most published protocols outlining phage isolation techniques focus on the identification and/or characterization of strictly lytic phages. Here, a simple, adapted protocol specific for the isolation of non-lytic, filamentous phages is described. This method can be used in laboratories with access to basic microbiological equipment. Briefly, the adapted protocol steps involve:

• Phage enrichment with F-pilus expressing bacterial host and sewage filtrate
• Dilution and agar overlay plating of enriched culture
• Selection of hazy, turbid plaques followed by screening via PCR

## Graphical abstract

**Figure:**
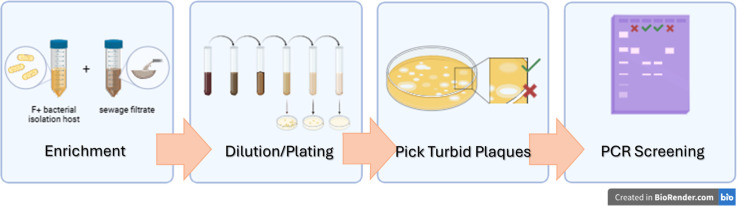

**Specifications table**

**Table:** 

**Subject area**	Biochemistry, Genetics and Molecular Biology
**More specific subject area**	Bacteriophage isolation
**Name of your method**	Isolation of F-specific Inovirus filamentous phages from environmental sewage
**Name and reference of original method**	*Detection of bacteriophages:* S. T. Abedon, “Detection of Bacteriophages: Phage Plaques,” in *Bacteriophages*, D. R. Harper, S. T. Abedon, B. H. Burrowes, and M. L. McConville, Eds., Cham: Springer International Publishing, 2021, pp. 507–538. doi: 10.1007/978–3–319–41,986–2_16. *Bacteriophage enrichment:* R. Van Twest and A. M. Kropinski, “Bacteriophage Enrichment from Water and Soil,” in *Bacteriophages: Methods and Protocols, Volume 1: Isolation, Characterization, and Interactions*, M. R. J. Clokie and A. M. Kropinski, Eds., Totowa, NJ: Humana Press, 2009, pp. 15–21. doi: 10.1007/978–1–60,327–164–6_2. *Enumeration of bacteriophages using double agar overlay:* A. M. Kropinski, A. Mazzocco, T. E. Waddell, E. Lingohr, and R. P. Johnson, “Enumeration of Bacteriophages by Double Agar Overlay Plaque Assay,” in *Bacteriophages: Methods and Protocols, Volume 1: Isolation, Characterization, and Interactions*, M. R. J. Clokie and A. M. Kropinski, Eds., Totowa, NJ: Humana Press, 2009, pp. 69–76. doi: 10.1007/978–1–60,327–164–6_7. *Agar layer method for phage production:* M. Swanstrom and M. H. Adams, “Agar Layer Method for Production of High Titer Phage Stocks,” *Exp. Biol. Med.*, vol. 78, no. 2, pp. 372–375, Nov. 1951, doi: 10.3181/00,379,727–78–19,076.
**Resource availability**	0.22 µm Whatman syringe filters, saline-magnesium (SM) buffer (50 mM Tris-HCl (pH 7.5) containing 100 nM NaCl and 8.1 mM MgSO_4_), LB-Lennox media with 1.5 % agar, LB-Lennox broth, 0.4 % molten LB agar supplemented with 2 mM CaCl_2,_ GoTaq® master mix, agarose, GelRed®, Axygen UV-gel documentation system

## Background

First discovered over 60 years ago, the F-specific filamentous (Ff) family of phages (comprising f1, fd and M13 phages) are neither lytic nor temperate, but rather exhibit a chronic infection-life cycle [1]. Upon binding to the F-pilus of *Escherichia coli* cells, pilus retraction allows for secondary receptor binding followed by internalization and uncoating of the phage genome [2]. Once the genome is uncoated, transcription and translation begin and more intriguingly, the chronic infection cycle is established with a double-stranded, replicative form (RF) genome within the cytoplasm [2]. This unique feature serves as a template to generate genomic infective form (IF) ssDNA, to be packaged into continuously budding phage particles released from the chronically-infected cell [2]. The non-enveloped, Ff phages are characterized by their genome synteny [3], or conserved gene order, which allows them to be quickly identified through polymerase chain reaction (PCR) analysis. Furthermore, these filamentous phages do not form clear plaques in soft agar, but rather zones resembling temperate phage-plaques characterized by hazy, turbid appearances, due to the relative growth restriction and decrease in cell density imparted on their bacterial host [4,5]. Their approximately 6.4 kb genome encodes six non-structural proteins and five structural proteins (gpVII, gpIX, gpVIII, gpIII and gpVI), of which the gpIII minor capsid protein and gpVIII major capsid protein have been most commonly engineered to display exogenous peptide sequences through phage display [2]. This technique has been used for multiple downstream applications, including but not limited to: construction of peptide display libraries, epitope mapping, study of protein-protein and protein-ligand interactions, therapeutic and vaccine strategies, nanomaterial design, and biosensor development [2,[6], [7], [8]]. Due to their ubiquity in environments in which *E. coli* thrive and their multitude of uses, Ff phage isolation from the environment is a critical first step in subsequent biological research and biotechnological product development.

Phage isolation protocols commonly utilize the soft-agar overlay plating method after environmental sample enrichment which largely targets the isolation of lytic phages, characterized by the appearance of clear plaques in the agar [5,[9], [10], [11]]. Here, an adapted protocol based on the same soft-agar overlay method of phage isolation is described for the specific purpose of isolating filamentous phages from environmental samples. This approach is intended to provide an accessible and cost-effective source of filamentous phages while broadening the genetic and functional repertoire available to researchers. Environmental isolation of Ff phages further eliminates the costs associated with purchasing commercial samples and simultaneously enables one to produce high-titred phage preparations, avoiding intellectual property restrictions that may limit downstream experimental use. Establishing these methods in the laboratory reduces limitations for future applications of filamentous phages in nanotechnology and therapeutic development.

## Method details

*Preparation and enrichment of raw sewage samples.* Environmental filtrates were prepared by centrifuging raw sewage samples at 5,000 x g for 30 minutes to pellet particulate matter. Supernatants were passed through 0.22 µm Whatman syringe filters (VWR, Mississauga, Canada) and stored at 4°C. Fresh overnight cultures of *E. coli* K37:HFr (ATCC 33626) were inoculated into a mixture of: 5 mL of 2X concentrated LB-Lennox broth (Fisher Scientific, Ottawa, Canada) supplemented with 4 mM CaCl_2_(Fisher Scientific, Ottawa, Canada) and 5 mL of the environmental filtrate. Samples were incubated at 37°C for 48 hours shaking at 50 rpm. Following incubation, samples were centrifuged for 30 minutes at 5,000 x g to pellet cells, and supernatants were passed through 0.22 µm Whatman syringe filters and stored at 4°C.

**Fig. 1:**
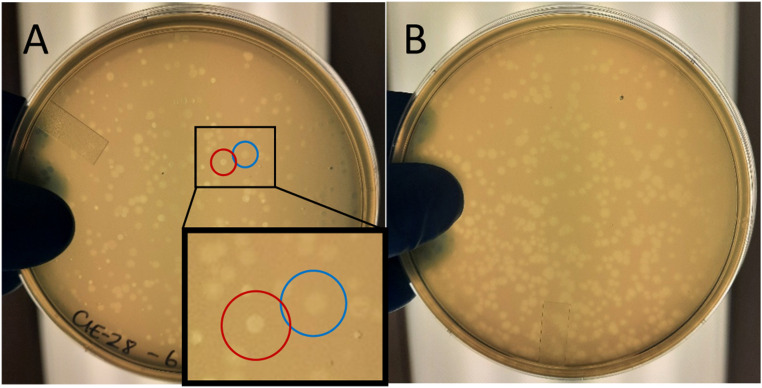
A; Double agar overlay plating of environmental phage enrichment indicated a mixed population of plaque morphologies (10^–6^ dilution is shown). Inset depicts enlarged plaques for visualization purposes. Red circles indicate lytic plaque morphology with clearing of bacterial lawn in the centre. Blue circles indicate hazy, turbid plaque morphology typical of filamentous phage plaquing. B; Double agar overlay plating of clonal population of turbid plaques as a result of three rounds of plaque purification (10^–6^ dilution shown).

*Double agar overlay method for isolation of phage enrichment.* A fresh overnight culture of *E. coli* K37:HFr (ATCC 33626) was inoculated into LB-Lennox broth at a ratio of 1:100 and incubated at 37°C while shaking at 150 rpm until early log phase (O.D._600_ = 0.3–0.5) was achieved. Ten-fold serial dilutions of the enriched and filtered environmental preparations were prepared in saline-magnesium (SM) buffer (50 mM Tris-HCl (pH 7.5) (VWR, Missisauga, Canada) containing 100 mM NaCl (Sigma Aldrich, Oakville, Canada) and 8.1 mM MgSO_4_ (Fisher Scientific, Ottawa, Canada)) to dilutions ranging from 10^–4^ to 10^–8^. To 3 mL of 0.4 % molten LB-Lennox agar (Fisher Scientific, Ottawa, Canada) supplemented with 2 mM CaCl_2_, 200 µL of early log phase *E. coli* K37:HFr (ATCC 33626) and 100 µL of each dilution of environmental enrichments (10^–4^ to 10^–8^) were combined, then the mixture was poured evenly on bottom agar plates containing LB-Lennox media with 1.5 % agar. Overlay plates were incubated at 37°C overnight to allow for visualization of isolated plaques (Fig. 1A).

*Filamentous plaque purification.* Five individual, non-lytic, hazy, turbid plaques were aseptically picked from the overlay plates and resuspended in 50 µL of sterile SM buffer. Each plaque suspension was serially diluted to 10^–6^ in SM buffer and plated using the double agar overlay method. Purification was repeated three times until a clonal population of uniform, hazy plaques was visualized on each overlay plate (Fig. 1B). Each isolated plaque was stored at 4°C in SM buffer.

**Fig. 2:**
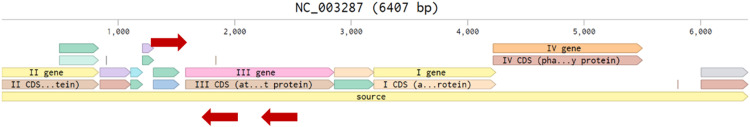
Linear representation of *Inovirus M13* (GenBank accession NC_003287) genome with annotated genes indicated primer binding sites (red arrows). The designed primer set amplifies two overlapping regions spanning the pVIII and pIII genes of M13-like phages. The reverse primer binds two regions, indicated by two reverse, red arrows, resulting in amplification of an 897 bp fragment and a 411 bp fragment.

**Fig. 3:**
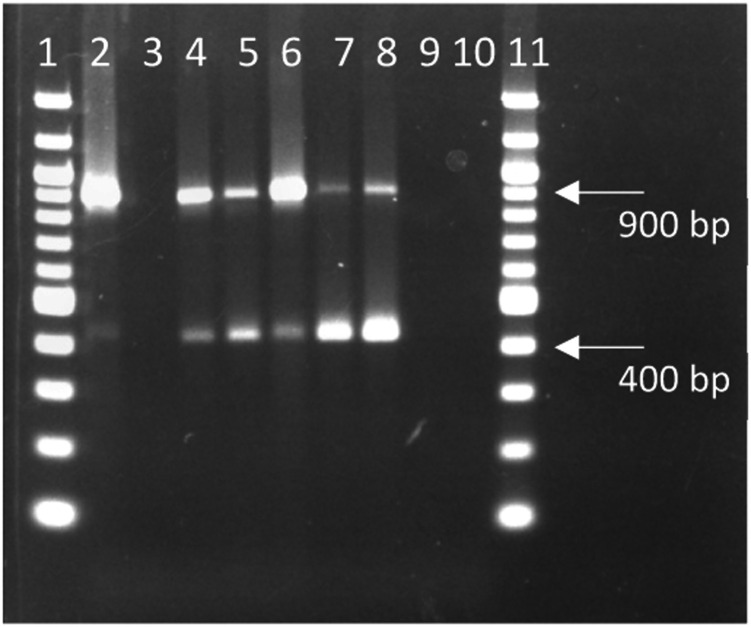
PCR confirmation of filamentous phage identity through amplification of 897 bp and 411 bp section of phage genome of positive control (M13 phage ATCC 15669-B1), negative bacterial control (*E. coli* K37:HFr ATCC 33626) and isolated plaque picks. Lane 1 and 11: 100 bp ladder; Lane 2: M13 plaque; Lane 3: bacterial control; Lane 4–8: isolated phage plaques 1–5; Lane 9: SM control reaction; Lane 10: nuclease-free water. Reactions were loaded into a 2 % agarose gel and run at 90 V for 60 minutes before staining in 1X GelRed® and visualized using an Axygen UV-gel documentation system.

*PCR confirmation of presence of filamentous phages.* Purified plaques were subjected to PCR amplification using newly designed primers specific to regions within the pVIII and pIII genes of the *Inovirus M13* genome (Table 1, Fig. 2). For each reaction, 5 µL of each plaque pick in SM buffer was combined with 12.5 µL of GoTaq® master mix (Promega, Madison, USA), 0.5 µL of each 10 µM forward and 10 µM reverse primer stock (Table 1) and 6.5 µL of nuclease-free water following the manufacturer’s recommendations with an annealing temperature of 65°C for 30 cycles. The positive control reaction included a plaque pick of M13 phage (ATCC 15669-B1) in SM buffer. Additional negative PCR control reactions included an *E. coli* K37:HFr (ATCC 33626) bacterial control in SM buffer, and SM buffer alone. After PCR, samples were loaded into a 2 % agarose gel and subjected to electrophoresis at 90 V for 60 minutes. The gel was then stained with 1X GelRed® (VWR, Mississauga, Canada) and visualized using an Axygen UV-gel documentation system (Fig. 2). PCR screening of M13 phage (ATCC 15669-B1) amplified two expected DNA regions of 897 bp and 411 bp in length (Fig. 3) using a single forward and reverse primer set (Table 1). The bacterial control and SM buffer control reaction yielded no DNA amplification. Five phages that were isolated through the agar overlay method were screened by plaque PCR resulting in DNA band sizes (spanning gpVIII to gpIII) corresponding to a similar banding pattern to that of the positive control M13 phage (Fig. 3). These regions are highlighted in the *Inovirus M13* (formally known as *Enterobacteria phage M13* (accession MC_003287)) genome sequence (Fig. 2).

**Table 1:** Primer sequences designed for PCR screening of purified phage plaques.

Primer Name	Primer Sequence (5′-3′)	Amplicon Size (bp)	Annealing Temperature (°C)
p8p3 FWD	atgcgtgggcgatggttgttgt	897 and 411	65 °C
p8p3 REV	tcagagccaccaccctcagagc	897 and 411	65 °C

## Method validation

*Confirmation of PCR screening by sequencing*. In 20 mL of LB-Lennox broth, 5 µL of each PCR-positive purified plaque was combined with 200 µL of log phase *E. coli* K37:HFr (ATCC 33626). Cultures were incubated for 16 hours at 37°C, shaking at 100 rpm. The next day, cultures were centrifuged at 3,000 x g for 20 minutes at 4°C to pellet cells. Supernatants were passed through 0.22 µm Whatman syringe filters, added to Amicon Ultra-4 100 kDa centrifugal filters (Millipore-Sigma, Oakville, Canada) and centrifuged at 4,000 x g for 20 minutes at 4°C, until the entire volume of supernatant was passed. Filters were washed twice with 10 mL SM buffer, before final resuspension in 1 mL of sterile SM buffer and filter sterilization through 0.22 µm Whatman syringe filters. To 400 µL of each phage preparation, 10 µL of DNAseI (2000 Units/mL) (New England BioLabs (NEB), Whitby, Canada) and 10 µL of RNAse If (50,000 Units/mL) (NEB, Whitby, Canada) were added and the mixtures were incubated for 1 hour at 37°C. Following incubation, 40 µL of Proteinase K (800 Units/mL) (NEB, Whitby, Canada) and 2 % sodium dodecyl sulfate (SDS) (Sigma Aldrich, Oakville, Canada) were added, incubated at 56°C for 1 hour followed by on-ice incubation for 5 minutes. To each reaction mixture, 400 µL of undiluted phenol:chloroform:isoamyl alcohol (Sigma Aldrich, Oakville, Canada) was added and each tube inverted for 5 minutes at room temperature. Tubes were then centrifuged at 13,000 x g for 5 minutes to separate the upper aqueous phase, then the entire volume (440 µL) from each tube was removed and placed into a clean microcentrifuge tube. To each tube, 0.1 volume (44 µL) of 3 M sodium acetate (Sigma Aldrich, Oakville, Canada) and 0.7 volume (308 µL) of 100 % ice-cold isopropanol (Sigma Aldrich, Oakville, Canada) were added and solutions were placed at -20°C for 1 hour. Solutions were then centrifuged at 17,900 x g for 10 minutes at 4°C, supernatants were removed and pellets were washed with ice-cold 70 % ethanol. Pellet-containing solutions were centrifuged again at 17,900 x g for 10 minutes at 4°C, supernatants were discarded then DNA pellets were air-dried at room temperature for 20 minutes. Pellets were resuspended in sterile, nuclease-free water and subjected to Illumina short-read sequencing (Donnelly Centre, University of Toronto).

*Illumina sequence assembly parameters*. Raw reads were assembled using the BV-BRC online SPAdes genome assembler v4.0.0 with minimum contig coverage threshold set at 50.0 and minimum contig length threshold set at 300 for each set of raw read sequencing files. Assembled genomes were then subjected to nucleotide BLAST analysis (NCBI Blast v2.16.0) to verify sequence identity to known Ff phage full genomes.

*Identification of filamentous phages.* Sequencing analysis indicated the unknown phage samples had genome sizes of 6485 bp or 6489 bp in length (Table 2). Nucleotide BLAST (BLASTn) analysis identified two phages with 99.66 % identity to *Enterobacteria phage FfIris* (accession PP430148.1; 6408 bp), two phages with 98.51 % identity to *Enterobacteria phage FfLavender* (accession PP430149.1; 6412 bp) and one phage with 98.99 % identity to *Escherichia phage fd* strain 478 (accession NC_025824.1; 6408 bp) (Table 2).

**Table 2:** Sequencing and BLASTn analysis results of five isolated phages.

Sample ID	Genome Length (bp)	Closest BLAST identity (genome length in bp)	Percent identity (%)	GenBank Accession Number
Phage 1	6485	*Enterobacteria phage FfIris* (6408)	99.66	PP430148.1
Phage 2	6485	*Enterobacteria phage FfIris* (6408)	99.66	PP430148.1
Phage 3	6485	*Escherichia phage fd* strain 478 (6408)	98.99	NC_025824.1
Phage 4	6489	*Enterobacteria phage FfLavender* (6412)	98.51	PP430149.1
Phage 5	6489	*Enterobacteria phage FfLavender* (6412)	98.51	PP430149.1

VIRIDIC comparative sequence analysis [12] determined that both Phages 1 and 2 were identical (Supplementary Figure 1). Phages 4 and 5 were also determined to be identical. The heat map generated from VIRIDIC analysis determined relative sequence similarities to one another and to the *Inovirus M13* genome (GenBank accession NC_003287) (Supplementary Figure 1).

## Limitations

None.

## Supplementary material *and/or* additional information

VIRIDIC comparative analysis [12] was performed on the genomic sequences generated from phage isolation and the output heat map is attached in the accompanying file (Supplementary Figure 1).

## Ethics statements

This work was fully supported by Cytophage Technologies Inc., a publicly traded company based in Oak Bluff, MB, Canada.

## CRediT authorship contribution statement

**Natasha Theriault:** Conceptualization, Methodology, Validation, Formal analysis, Investigation, Writing – original draft, Visualization, Project administration. **Bradley W.M. Cook:** Writing – review & editing, Supervision. **Steven S. Theriault:** Supervision, Funding acquisition. **Deborah A. Court:** Writing – review & editing, Supervision.

## Declaration of competing interest

The authors declare that they have no known competing financial interests or personal relationships that could have appeared to influence the work reported in this paper.

## Data Availability

No data was used for the research described in the article.

## Appendix Supplementary materials

Image, application 1
