# Supplementary material for: Isolation of F-specific Inovirus filamentous phages from environmental sewage samples
Source: MethodsX. 2025 Oct 30;15:103701. doi: 10.1016/j.mex.2025.103701 (PMC12648497; doi:10.1016/j.mex.2025.103701)
Supplement: Supplementary file 1 [file mmc1.docx]

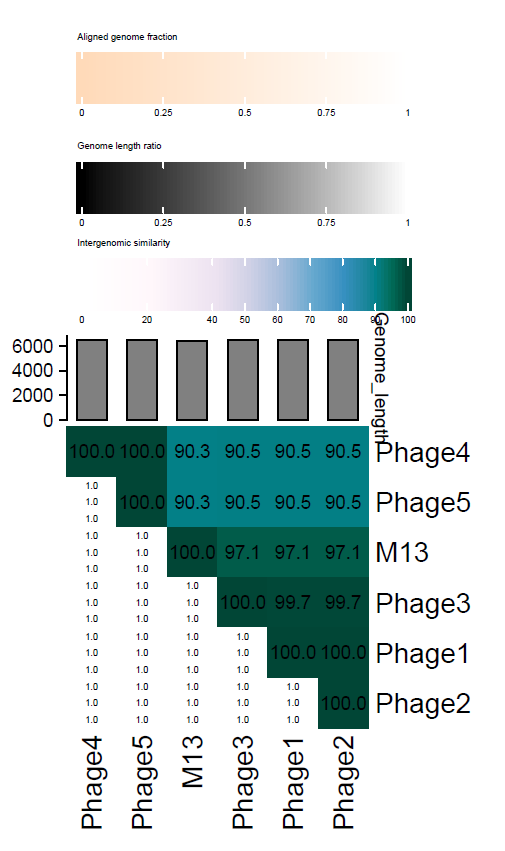


**Supplementary Figure 1.** Heat map generated from VIRIDIC comparative genome analysis *of Inovirus M13* genome (GenBank accession NC_003287) and isolated phage genomes indicating relatedness. Species threshold was set at 95 and genus threshold was set at 70. Colour gradient indicates intergenomic similarities according to the included legend.
